# Community health worker-led ART delivery improved scheduled antiretroviral drug refill among men who have sex with men in Lagos State, Nigeria

**DOI:** 10.1093/inthealth/ihaa021

**Published:** 2020-06-01

**Authors:** Olujuwon Ibiloye, Patrick Akande, Jwanle Plang, Franklin Emerenini, Temiwoluwa Omole, Olusola Osindero, Tom Decroo

**Affiliations:** APIN Public Health Initiatives 10, Ndagi Mamudu Close, Jabi district, Abuja, Nigeria; Instituut voor Tropische Geneeskunde 155, Nationalestraat, 2000 Antwerp, Belgium; APIN Public Health Initiatives 10, Ndagi Mamudu Close, Jabi district, Abuja, Nigeria; APIN Public Health Initiatives 10, Ndagi Mamudu Close, Jabi district, Abuja, Nigeria; APIN Public Health Initiatives 10, Ndagi Mamudu Close, Jabi district, Abuja, Nigeria; APIN Public Health Initiatives 10, Ndagi Mamudu Close, Jabi district, Abuja, Nigeria; APIN Public Health Initiatives 10, Ndagi Mamudu Close, Jabi district, Abuja, Nigeria; Instituut voor Tropische Geneeskunde 155, Nationalestraat, 2000 Antwerp, Belgium; Research Foundation Flanders 5, Egmontstraat, 1000 Brussel, Belgium

**Keywords:** antiretroviral therapy, community health worker, HIV, men who have sex with men

## Abstract

**Background:**

Stigma affects access and treatment outcomes in men who have sex with men. We assessed the effect of novel community health worker-led antiretroviral therapy delivery (CLAD).

**Methods:**

A retrospective cohort study of routinely collected data was conducted. We used the *t*-test to compare the mean adherence to scheduled drug refill appointments before and after implementing CLAD.

**Results:**

The mean adherence to drug refill was 1.4 (±0.7 SD) of monthly scheduled refills before CLAD and 4.7 (±1.2 SD) of monthly refills in CLAD (*P* < 0.001).

**Conclusion:**

The CLAD model was more effective for drug refill appointments than a regular HIV clinic.

## Introduction

Globally, men who have sex with men (MSM) are disproportionately affected by HIV infection, are underserved and have limited access to quality HIV services. Nigeria counts an estimated 26 014 MSM, with an HIV prevalence of 23%,^1^ much higher than the national HIV prevalence of 1.5%.^2^ Health facilities providing care to the general population are often perceived as hostile by MSM, hampering access of MSM to HIV prevention, care and treatment services and negatively affecting antiretroviral treatment (ART) outcomes.^3^

To overcome such challenges the WHO recommends differentiated ART service delivery models that include key population (KP)-friendly community-based ART delivery.^4^ Previously described community health worker-led ART models did not target KPs.^5^

In Lagos State, in the regular healthcare facility, we observed a high rate of lost to follow-up among MSM. MSM were reportedly facing stigma and discrimination in the health facility. As a programmatic response, we shifted drug refill for MSM from the general clinic to the community relying on community health workers. This novel model was named community health worker-led ART delivery (CLAD). The objective of this paper is to evaluate the effect of the CLAD model on adherence to drug refill.

## Methods

A retrospective cohort study was conducted using data from MSM at the regular clinic (January–June 2018) and CLAD (July–December 2018).

### CLAD model

The CLAD model involved down-referral of HIV-positive MSM on ART to a KP-friendly community-based organization (CBO) for their monthly drug refill at the CBO's office or a hotspot venue (where MSM congregate for activities). The CBO obtained ART from the health facility. All clients enrolled into CLAD were on the same standardized first-line regimen. Lay health care worker (HCW), including adherence counselors, peer educators and community extension workers, refilled ART and offered counseling, symptom checks and condoms. MSM could ask a buddy to collect the refill (ART pick-up by proxy). Clients lost to follow-up were actively tracked through telephone calls. Patients were down-referred regardless of criteria, such as clinical stability, absence of opportunistic infections, adherence to treatment regimens and virological response.

Routinely collected program data were collected from patient files and registers. We calculated the *t*-test to compare the mean adherence to monthly scheduled drug refill appointments between 6 months at the general clinic (before CLAD) and 6 mo in CLAD.

## Results

By 30 June 2018, 93 MSM had initiated ART at the general clinic, 24 (25%) were active in care (had picked up drugs within the last 3 mo) and 69 (75%) were inactive.

All 24 active patients and 12 patients traced back to care from the inactive group were enrolled in the CLAD model. The mean age of 36 MSM in the CLAD model was 25 y (±4.4 SD). No patient had a recent viral load result.

In the general clinic and in CLAD, patients adhered to 22.4% (100/446) and 60% (108/180) of all scheduled drug pick-ups, respectively (Table 1). The mean adherence to drug refill was 1.4 (±0.7 SD) of monthly scheduled refills in the general clinic (before CLAD) and 4.7 (±1.2 SD) of refills in CLAD (*P* < 0.001). Of patients in CLAD, 75% (27/36) had a viral load and 74.1% (20/27) achieved viral load suppression.

**Table 1.** Drug pick-up in the general clinic and the community health worker-led ART delivery (CLAD) model

	General clinic drug refill				CLAD drug refill		
	Expected number of patients for drug refill^$^	Number of patients that came for refill	% Drug refill		Expected number of patients for drug refill	Number of patients that came for refill	% Drug pick-up
Jan	40	21	52.5	Jul	36	32	88.9
Feb	64	38	59.4	Aug	36	32	88.9
Mar	80	19	23.8	Sep	36	27	75.0
Apr	81	18	22.2	Oct	36	32	88.9
May	88	18	20.5	Nov	36	28	77.8
June	93	7	7.5	Dec	36	17	47.2
6 months	446	100	22.4	6 months	180	108	60.0

$ Cumulative number of men who have sex with men initiated on antiretroviral treatment in the general clinic.

## Discussion

There are few studies on community-based ART in Nigeria; however, to the best of our knowledge, this is the first community-based ART model to explore the use of lay HCW-led ART refill among MSM. The improved drug refill observed in the CLAD model is probably due to reduction of stigma and discrimination experienced by MSM in the health facility.^3^ In addition, MSMs were able to access their medications at their own convenience either at the CBO's office or hotspots, which we believe improved their adherence to drug refill appointment.

These early results of the CLAD model show the potential of a specialized client-centered approach to improve medication adherence among KPs and thus increase retention in care and viral load suppression. On the other hand, the 6-mo period overall drug pick-up was still low (60%).^6^ To enhance self-management, peer support and access to medical care, future modifications that we will evaluate will include multi-month drug dispensing, peer-led ART delivery and integration into the activities of an existing one-stop shop (OSS) clinic for KPs. The OSS clinic is a community-based HIV clinic, established strictly for KPs, managed by members of a medical outreach team and lay community actors.^7^ Meanwhile, the national HIV program could adopt lessons learnt from this model and evaluate the effect of the involvement of lay HCWs in HIV service provision to KPs, including community-based ART delivery.

This pilot study shows the reality of the KP HIV care program in one state in Nigeria. Formative research, including a cycle of piloting new approaches, evaluation and informed adaptation to care delivery, will be needed to control the HIV epidemic by 2030 targets,^8^ especially for vulnerable subgroups. The before-after design is a limitation to our study. A prospective cohort study is recommended to assess the effect of CLAD on retention-in-care and viral load suppression. Moreover, a substantial proportion of patients lost to follow-up from facility-based care were not traceable and thus not enrolled in CLAD, which may have resulted in selection bias. The short duration of the study period did not allow medium or long-term assessment of clinical outcomes.

In conclusion, this pilot study demonstrates that lay HCWs can be involved in ART delivery to MSM living with HIV and may inform similar initiatives.

## Authors’ statements

OI, PA and JP conceived and designed the study; OO, OI, PA, JP, FE and TO implemented the study; OI, TD and PA analyzed and interpreted data; TD, OI, PA, JP, FE, TO and OO contributed to draft writing, read and approved the final version.
